# Sexual orientation and self-reported mood disorder diagnosis among Canadian adults

**DOI:** 10.1186/1471-2458-13-209

**Published:** 2013-03-08

**Authors:** Basia Pakula, Jean A Shoveller

**Affiliations:** 1 School of Population and Public Health, University of British Columbia, Vancouver, British Columbia, Canada

**Keywords:** Mood disorders, Mental health, Sexual orientation, Sexual minority

## Abstract

**Background:**

The prevalence and correlates of mood disorders among people who self-identify as lesbian, gay or bisexual (LGB) are not well understood. Therefore, the current analysis was undertaken to estimate the prevalence and correlates of self-reported mood disorders among a nationally representative sample of Canadian adults (ages 18 to 59 years). Stratified analyses by age and sex were also performed.

**Methods:**

Using data from the 2007–2008 Canadian Community Health Survey, logistic regression techniques were used to determine whether sexual orientation was associated with self-reported mood disorders.

**Results:**

Among respondents who identified as LGB, 17.1% self-reported having a current mood disorder while 6.9% of heterosexuals reported having a current mood disorder. After adjusting for potential confounders, LGB-respondents remained more likely to report mood disorder as compared to heterosexual respondents (AOR: 2.93; 95% CI: 2.55-3.37). Gay and bisexual males were at elevated odds of reporting mood disorders (3.48; 95% CI: 2.81–4.31), compared to heterosexual males. Young LGB respondents (ages 18–29) had higher odds (3.75; 95% CI: 2.96–4.74), compared to same-age heterosexuals.

**Conclusions:**

These results demonstrate elevated prevalence of mood disorders among LGB survey respondents compared to heterosexual respondents. Interventions and programming are needed to promote the mental health and well being of people who identify as LGB, especially those who belong to particular subgroups (e.g., men who are gay or bisexual; young people who are LGB).

## Background

It is estimated that nearly one in five Canadian adults will experience a mental illness during their lifetime [1,2]. Mood disorders, such as depression and bipolar disorder, are among the most common mental illnesses in Canada [2], and pose potentially serious adverse effects for people’s social and work or school lives as well as for their general health and functioning [3]. Previous studies in the United States (US) have found that lesbian, gay, and bisexual (LGB) people in general, and LGB youth in particular, consistently report poorer mental health compared to the general population, including greater depression, stress, anxiety, substance use, and suicide [4-8]. These findings have been corroborated in recent studies in the United Kingdom where non-heterosexual orientation was found to be associated with elevated levels of mental disorders, self-harm and suicidality [9,10]. Few Canadian studies to date have examined associations between sexual orientation and mental health conditions (e.g., suicidality, depression, mood and anxiety disorders) in regional [11] and national [12,13] samples, noting poorer outcomes for LGB respondents.

A person’s sexual orientation has also been shown to be independently associated with mental health status, suggesting that the LGB people may face unique factors associated with being members of a sexual minority. For example, in one community-based study of adult lesbians and heterosexual women [7], non-heterosexual orientation was an independent predictor of depressive stress (a composite measure of depression indicators). In another community sample of lesbian and heterosexual women, sexual orientation also was a significant predictor of mental health utilization controlling for known confounders [14]. In two separate Canadian studies of gay and bisexual men [12] and lesbian and bisexual women [13], sexual orientation was independently associated with mood and anxiety disorders after controlling for smoking, BMI, and demographic characteristics.

The factors that appear to affect the mental health of LGB people are in large part similar to those affecting the mental health of heterosexual people; however, LGB people tend to be more likely to report experiencing stressful life events and anxiety [5,7,8], exposure to discriminatory behaviour [15,16], social isolation, low social supports and community belonging [5,17], and low mental health service utilization [14,18]. Unique factors noted in the literature include perceived discrimination [19], externalized and internalized homophobia [20,21], negative disclosure reactions [22,23], heterosexism and heteronormativity [24].

The majority of research on this topic has been conducted in the US [9], where, in many states, sexual minorities face relatively significant structural forms of discrimination (e.g., barriers to health care and health insurance; lack of power to make medical and legal decisions on behalf of an ill or deceased partner; lack of opportunity for same sex marriage by the federal government and most state governments) [25,26]. In Canada, a small number of studies examined the role of sexual orientation in mental health service utilization, and found that LGB people report higher unmet mental health service needs compared to non-LGB people [27,28]. However, empirical research on the prevalence of mental disorders among LGB Canadians remains scant, in particular as it pertains to understanding the possible causes of the elevated prevalence. With the exception of a series of studies on the health of LGB youth in British Columbia, Canada [11,29], this gap in evidence also pertains to the mental health of young people who identify as LGB. This study builds on existing studies in Canada to examine the prevalence and correlates of self-reported mood disorders, including effect modification by age and sex, in a nationally representative sample of LGB Canadian adults (ages 18 to 59 years). This is a step towards understanding possible mechanisms underlying the noted associations in the elevated prevalence of mood and other mental health disorders among LGB people [9] and in proposing appropriate interventions to addressing the observed inequities.

## Methods

### Data source

Data for the study were obtained from the 2007–2008 Canadian Community Health Survey. The CCHS is an ongoing, national, cross-sectional survey that collects information on health status, health care utilization and health determinants for the Canadian population. The CCHS is representative of approximately 98% of the Canadian population aged 12 and over living in private dwellings in the 121 health regions from all provinces and territories. Excluded from the sampling frame are individuals living on First Nations Reserves and Crown Lands, institutional residents (e.g., federal penitentiaries), full-time members of the Canadian Forces, and residents of extremely remote regions. Data are collected using computer-assisted in-person and telephone interviewing. Further details on the methodology of the 2007–2008 CCHS and its measures are reported by Statistics Canada [30]. Ethical approval for the use of the data was covered by the publicly available data clause of the University of British Columbia policy no. 89 on research and other studies involving human subjects.

### Study sample

The 2007–2008 CCHS included 131,959 respondents to all the survey questions over the two-year period. The sample in the present study was restricted to valid responses for the study outcome (self-reported mood disorder diagnosis) and primary explanatory variable (self-reported sexual orientation), for a total unweighted sample of 76,630. Excluded invalid responses were those who responded “don’t know,” did not state, or refused to answer the questions on the two primary variables of interest. In the CCHS, the primary explanatory variable was asked only of respondents ages 18 to 59; therefore the study sample is restricted to ages 18–59. Based on the sample size of 76,630 respondents, the study has 0.95 power (alpha=0.05/beta=0.2) to detect a difference in the prevalence of mood disorders by sexual orientation equal to 0.02.

### Study variables

The main outcome of the study was self-reported mood disorder diagnosis (yes/no) based on the survey question: “Do you have a mood disorder such as depression, bipolar disorder, mania or dysthymia?” Respondents were reminded to self-report conditions diagnosed by a health professional; however, they were not asked to elaborate on the type of mood disorder diagnosis or the time when the diagnosis occurred. The primary explanatory variable, self-reported sexual orientation (heterosexual/ LGB), was based on the question: “Do you consider yourself to be: (heterosexual, homosexual, that is lesbian or gay, or bisexual)?” The sexual orientation variable was dichotomized into two categories: heterosexual and lesbian/gay/bisexual (LGB).

Potential confounders of the relationship between mood disorders and sexual orientation were also examined in light of previous evidence in the peer-reviewed literature [4,5,7,17,24] suggesting their association with both the outcome and the primary explanatory variable. These included: sex, age, educational attainment, perceived life stress, sense of community belonging, and having a regular general practitioner (GP), which served as a proxy for health service utilization.

Age was derived from a continuous variable and grouped into three age categories (18–29, 30–39, 40–59). Perceived life stress was based on the question: “Thinking about the amount of stress in your life, would you say that most days are: (not at all stressful, not very stressful, a bit stressful, quite a bit stressful, or extremely stressful)?” Responses “not at all stressful,” “not very stressful” and “a bit stressful” were recoded as “not very stressful” and responses “quite a bit stressful” and “extremely stressful” were recoded as “very stressful.” Sense of community belonging was based on the question: “How would you describe your sense of belonging to your local community? Would you say it is: (very strong, somewhat strong, somewhat weak, or very weak)?” Responses “very strong” and “somewhat strong” were recoded as “strong” and responses “somewhat weak” and “very weak” were recoded as “weak.” The variables sex (male/ female); having a regular medical doctor (yes/ no); and educational attainment, which denoted the highest level of household educational attainment (less than secondary/ secondary/ other post-secondary/ post-secondary), preserved original survey question categories.

### Data analyses

Data analyses were conducted using SAS 9.3 (SAS Institute Inc., Cary, NC). To appropriately account for the CCHS sampling procedures [30], probability weights were applied in all analyses to produce unbiased estimates and variances that adjusted for the sampling strategy. Chi-square was used to examine bivariate relationships between self-reported mood disorder and the various correlates described above. The relationship between sexual orientation and mood disorder diagnosis was first examined using an unadjusted OR with a 95% confidence interval (CI). Next, OR and 95% confidence intervals adjusted for age and sex were calculated. After testing for collinearity (using Spearman’s rho) and interaction, a multivariable logistic regression model with all study variables was used to measure the presence and strength of the relationship between sexual orientation and mood disorders, while controlling for the effect of confounders. Adjusted ORs and corresponding 95% confidence intervals were calculated (α=0.05). Stratified analyses were conducted to explore effect modification by age and sex.

Two sets of missing data were explored to determine whether missing data were randomly distributed across study variables. First, all analyses were repeated on a sample restricted to valid responses for all variables (n=75,490). Second, age, sex, and outcome distributions were obtained for a sample with missing responses to the sexual orientation question (i.e., those who answered ‘don’t know,’ ‘refused,’ or ‘did not state’ a response recoded as ‘missing’) (n=3,211).

## Results

### Description of study population

Table 1 shows the distribution of all study variables by mood disorder diagnosis. Of the total sample, 7.1% (n=6546) reported a mood disorder diagnosis. Respondents self-identifying as LGB were significantly more likely to report mood disorder diagnosis, with 17.1% of LGB reporting a mood disorder compared to 6.9% of heterosexuals (p<0.0001, 1df)*.* Canadians with mood disorders were more likely to be female (9.6% vs. 4.7%) (p<.0001, 1df), report very high levels of life stress (12.6% vs. 5.2%) (p<.0001, 1df), have a weak sense of community belonging (9.1% vs. 5.9%) (p<.0001, 1df), have a regular GP (7.8% vs. 4.2%) (p<.0001, 1df), be in the older age groups (p<.0001, 2df), and have lower levels of education (p<.0001, 3df).

**Table 1:** Distribution of study sample by mood disorder diagnosis

**Characteristics**	**Mood disorder**	
	**Yes**	**No**
	**n=6546 (7.1%)**	**n=70084 (92.9%)**
*Sexual orientation		
Heterosexual	6232 (6.9%)	68768 (93.1%)
LGB	314 (17.1%)	1316 (83.0%)
*Sex		
Male	2112 (4.7%)	33329 (95.3%)
Female	4434 (9.6%)	36755 (90.5%)
*Self-perceived life stress		
Not very high	3571 (5.2%)	54469 (94.8%)
Very high	2960 (12.6%)	15451 (87.4%)
*Community belonging		
Strong	3293 (5.9%)	45596 (94.1%)
Weak	3201 (9.1%)	23813 (90.9%)
*Regular GP		
Yes	5786 (7.8%)	56457 (92.2%)
No	757 (4.2%)	13575 (95.8%)
*Age		
18–29	1190 (5.8%)	16609 (94.2%)
30–39	1415 (6.5%)	16978 (93.5%)
40–59	3941 (8.1%)	36497 (91.9%)
*Education		
Less than secondary	1034 (10.4%)	8139 (89.6%)
Secondary	1094 (7.1%)	12512 (92.9%)
Other post-secondary	687 (8.3%)	6079 (91.7%)
Post-secondary	3706 (6.4%)	43135 (93.6%)

*Note*. Chi-square test: * p<.0001.

A total of 2.1% respondents (2.0% female and 2.2% male) self-identified as LGB. Chi-square analyses were performed to examine differences in the distribution of all study variables across the primary explanatory variable (see Table 2). Compared to heterosexuals, LGB respondents were more likely to report: higher levels of self-perceived life stress (28.6% vs. 25.8%) (p=0.0123, 1df), a weak sense of community belonging (44.1% vs 38.9%) (p<0.0001, 1df), not having a regular GP (21.9% vs. 18.6%) (p=0.008, 1df), being in the younger age groups (p<0.0001, 2df), and being in the post secondary education groups (p<0.0001, 3df). No collinearity was observed between stress, community belonging, and having a regular GP (all r< 0.2).

**Table 2:** Distribution of study sample by sexual orientation

**Characteristics**	**Sexual orientation**	
	**Heterosexual**	**LGB**
	**n=75000 (97.92%)**	**n=1630 (2.72%)**
*Mood disorder		
Yes	6232 (6.92%)	314 (17.05%)
No	68768 (93.08%)	1316 (82.95%)
**Sex**		
Male	34648 (49.73%)	793 (50.24%)
Female	40352 (50.27%)	837 (49.76%)
**Self-perceived life stress		
Not very high	56879 (74.22%)	1161 (71.44%)
Very high	17946 (25.78%)	465 (28.56%)
*Community belonging		
Strong	47963 (61.16%)	926 (55.86%)
Weak	26327 (38.84%)	687 (44.14%)
**Regular GP		
Yes	60944 (81.45%)	1299 (78.15%)
No	14004 (18.55%)	328 (21.85%)
*Age		
18–29	17337 (27.0%)	462 (32.71%)
30–39	18064 (22.9%)	329 (19.37%)
40–59	39599 (50.11%)	839 (47.92%)
*Education		
Less than secondary	9007 (10.11%)	166 (7.42%)
Secondary	13363 (17.32%)	243 (14.48%)
Other post-secondary	6562 (9.65%)	204 (14.47%)
Post-secondary	45831 (62.91%)	1010 (63.63%)

*Note*. Chi-square test: * p<.0001, ** p<.05.

### Association between sexual orientation and mood disorders

LGB Canadians were found to have 2.76 higher odds of mood disorder diagnosis compared to heterosexuals (95% CI: 2.42–3.16) in the unadjusted model. In the fully adjusted model, LGB had 2.93 higher odds of mood disorder diagnosis compared to heterosexuals (95% CI: 2.55–3.37). Table 3 summarizes weighted unadjusted and adjusted odds ratios for the association between sexual orientation and mood disorders.

**Table 3:** Adjusted and unadjusted odds ratios for mood disorder and sexual orientation

**Characteristic**	**Unadjusted (n=76,630)**		**Adjusted**^**1 **^**(n=76,386)**	
	**OR**	**95% CIs**	**OR**	**95% CIs**
**Sexual orientation**				
LGB	2.76*	2.42–3.16	2.93*	2.55–3.37
Heterosexual	1.0		1.0	
**Sex**				
Female	2.14*	2.02–2.27	2.03*	1.97–2.15
Male	1.0		1.0	
**Age**				
18–29	0.70*	0.65–0.75	0.72*	0.67–0.77
30–39	0.79*	0.74–0.85	0.85*	0.79–0.91
40–59	1.0		1.0	
**Education**				
Less than secondary	1.69*	1.56–1.84	1.92*	1.77–2.10
Secondary	1.32*	1.21–1.45	1.51*	1.37–1.65
Other post-secondary	1.11*	1.03–1.20	1.23*	1.14–1.33
Post-secondary	1.0		1.0	
**Regular GP**				
Yes	1.91*	1.75–2.08	1.75*	1.60–1.92
No	1.0		1.0	
**Community belonging**				
Weak	1.61*	1.53–1.70	1.61*	1.52–1.70
Strong	1.0		1.0	
**Self-perceived life stress**				
Very high	2.61*	2.46–2.76	2.50*	2.36–2.65
Not very high	1.0		1.0	

*Note*. Wald test: * p<.0001.

^1^ Adjusts for age group, sex, education, having a regular GP, community belonging, and life stress.

### Effect modification by sex and age

Effect modification by sex and age was explored in separate stratified analyses. Table 4 shows the results of these analyses, reporting the odds ratios for the association between sexual orientation and mood disorders by age group and sex. Lesbian and bisexual women had 2.60 higher odds of mood disorders (95% CI: 2.17–3.12) compared to heterosexual females; gay and bisexual males had 3.5 higher odds of mood disorders (95% CI: 2.81–4.31) compared to heterosexual males. LGB respondents aged 18–29 were found to have the highest odds for mood disorders at 3.75 (95% CI: 2.96–4.74) compared to heterosexual respondents in the same age group.

**Table 4:** Adjusted odds ratios for mood disorders and sexuality stratified by sex and age

**Characteristic**	**Sexual orientation**	**Adjusted**^**1**^	
		**OR**	**95% CIs**
**Sex**^**2**^			
Male	GB	3.48*	2.81–4.31
	Heterosexual	1.0	
Female	LB	2.60*	2.17–3.12
	Heterosexual	1.0	
**Age**^**3**^			
18–29	LGB	3.75*	2.96–4.74
	Heterosexual	1.0	
30–39	LGB	2.68*	1.91–3.76
	Heterosexual	1.0	
40–59	LGB	2.53*	2.07–3.09
	Heterosexual	1.0	

*Note*. Wald test: * p<.0001.

^1^Adjusts for age group, sex, education, having a regular GP, community belonging, and life stress.

^2^Sex: female (n=41,189); male (35,441).

^3^Age: 18–29 (n=17,799); 30–39 (n=18,393); 40–59 (n=40,438).

### Missing data

Results of regression analyses on a sample without missing values (n=75,490) compared to the study sample with missing values (n=76,386) yielded nearly identical odds ratios, with slightly wider confidence intervals at the upper end. Age, sex, and outcome distributions for those with missing responses to the sexual orientation question (n=3,211) showed that missing respondents were more likely to be male (p=0.0064, 1df) and be in the older age groups (p=0.0874, 2df) compared to heterosexual and LGB respondents. These respondents had higher rates of mood disorders compared to heterosexuals (9.5% vs. 6.9%), but lower compared to LGB (vs. 17.1%) (p<0.0001, 2df).

## Discussion

Three key findings emerge from this study regarding the association between sexual orientation and self-reported mood disorder diagnosis. First, lesbian, gay and bisexual Canadians reported greater odds of mood disorder diagnoses compared to their heterosexual counterparts. This effect persisted, and in fact increased slightly, after controlling for levels of stress, community belonging, having a GP, and socio-demographic characteristics. Second, in stratified analyses, both males and females in the LGB group had elevated odds of mood disorders compared to heterosexual males and females, with the highest odds noted among gay and bisexual males. Third, while increased odds of mood disorders for LGB respondents were observed across all age groups, the highest odds ratios were noted among young LGB aged 18–29, with nearly quadruple odds compared to their same-age heterosexual counterparts.

The results of the study build on growing evidence of increased prevalence of self-reported mood disorders for LGB Canadians. The findings are consistent with international studies reporting poorer mental health outcomes, such as depression, anxiety disorders, and suicide, among LGB people [4,5,7-10,31]. Although it has been suggested that factors related to marginalization and stigma (e.g., homophobia, heterosexism, disclosure reactions, and sexual discrimination) [19-23] contribute to elevated mood disorder prevalence in the LGB community, the exact nature of the mechanisms and pathways that shape mental health in these communities remain poorly understood.

The stratified findings of effect modification by age and sex are consistent with existing literature on the importance of factors, such as sex, age, race, and sexual orientation, in understanding health inequalities in Canada [32]. The stratified results are also consistent with literature reporting poorer mental health outcomes of young LGB people [6,8,33]. These findings point to the possible mechanisms underlying the independent effect of sexual orientation on mood disorder diagnosis, after controlling for known confounders. A number of studies discuss the mental health implications as young people navigate the stages of sexual identity development [22,34]. For example, in one US study, negative social reactions to disclosing one’s LGB identity were associated with subsequent substance use, while positive reactions were thought to serve as stress-buffering or resilience-building for LGB people [23]. The current findings show substantially higher odds of mood disorders among gay and bisexual males (compared to heterosexual males), which point to important sex- and gender-based considerations regarding the mental health inequalities experienced by LGB people.

The current study has several limitations and strengths. The self-reported nature of the data may be subject to recall and social desirability biases as well as the subjective interpretation of the questions. While respondents were asked to report mood disorders diagnosed by a health professional, this primary outcome was self-reported and the date of the diagnosis is unknown. The proportion of LGB Canadians is also likely underestimated in the CCHS. The CCHS asks about the sexual orientation of people aged 18–59 only, thus excluding adolescents and seniors. Based on estimates from the US, the proportion of people who choose to self-identify as LGB is approximately 2% while those who report same sex behaviour ranges from 5–7% [35]. Similarly, about 2.7% of CCHS respondents self-identified as LGB. Furthermore, existing literature suggests that self-identification or the act of disclosure (“coming out”) is an indication of self-acceptance, reducing stress associated with concealing or denying one’s sexual orientation [23,34]. Survey respondents who chose to self-identify as LGB in the CCHS may therefore be more likely to report better mental health outcomes than those who did not self-identify (i.e., remain ‘closeted’), leading to an underestimation of the true effect between sexual orientation and mood disorders in the study. Additionally, although the study is strengthened by the use of a large, population-based survey sample, the sample size of LGB respondents impaired our ability to conduct additionally stratified analyses (e.g., separate effects for gay, lesbian, and bisexual people). In particular, the study could not examine multiple intersecting vulnerabilities, such as race, gender identity, or immigration status, which some other studies have suggested may also be important contributors to mental health outcomes [32,36-38]. Finally, the cross-sectional design of the CCHS limits the ability to assess causal relationships, although we acknowledge that a fulsome investigation of these mechanisms may demand the use of an array of methods (qualitative and quantitative) to facilitate more nuanced explanations, particularly amongst hard-to-reach populations [39].

## Conclusions

These findings point to mental health needs among LGB people and highlight the importance of accounting for particular risk factors that are associated with being a member of a stigmatized sexual minority. In light of the prevalence and potential severity of mental health problems, there is an urgent need to better understand – and act upon – the conditions and practices that contribute to the likelihood of LGB people experiencing mood disorders. Additional research to identify the pathways of risk and resilience is required in order to point towards possible policy and programming interventions, including novel efforts to intervene to prevent the emergence of mood disorders (e.g., interventions to help with stressful life events; interventions designed tailored to address social stigma, including heterosexism and homophobia). Finally, the scope of the problem also demands responses within the mental health care system to ensure that the mental health treatment needs of LGB are responded to appropriately (e.g., LGB-friendly approaches; low-threshold service provision).

## Competing interests

The authors declare that they have no competing interests.

## Authors’ contributions

BP conceived of the study design, and was responsible for the accuracy of statistical analyses and the composition of manuscript. JAS wrote and reviewed manuscript drafts, and provided significant scientific input to the interpretation of the data and results. Both authors read and approved the final version of the manuscript. Research was conducted between October 2011-March 2012.

## Pre-publication history

The pre-publication history for this paper can be accessed here:

http://www.biomedcentral.com/1471-2458/13/209/prepub
